# The association between *Helicobacter pylori* seropositivity and risk of new-onset diabetes: a prospective cohort study

**DOI:** 10.1007/s00125-017-4465-2

**Published:** 2017-10-30

**Authors:** Mengge Zhou, Jing Liu, Yue Qi, Miao Wang, Ying Wang, Fan Zhao, Yongchen Hao, Dong Zhao

**Affiliations:** Department of Epidemiology, Beijing Anzhen Hospital, Capital Medical University, Beijing Institute of Heart, Lung and Blood Vessel Diseases, the Key Laboratory of Remodelling-Related Cardiovascular Diseases, Ministry of Education, No. 2 Anzhen Road, Chaoyang District, Beijing, 100029 China

**Keywords:** Diabetes, *Helicobacter pylori*, Infection, Prospective cohort study, Seropositivity

## Abstract

**Aims/hypothesis:**

Previous studies have suggested a possible connection between *Helicobacter pylori* (*H. pylori*) infection and diabetes risk. However, prospective studies examining direct associations between these two factors are relatively lacking. In this prospective cohort study, we aimed to evaluate the association between *H. pylori* infection and risk of developing diabetes.

**Methods:**

We performed a population-based prospective study, recruiting participants aged 45–74 years and without diabetes from the Chinese Multi-provincial Cohort Study in 2002, with a 10 year follow-up to investigate development of diabetes. *H. pylori* serostatus was determined by measuring serum *H. pylori* antibodies. *H. pylori* seropositivity was defined as the antibody concentration ≥ 10 U/ml. To examine the association between *H. pylori* seropositivity and diabetes risk, modified Poisson regression was performed.

**Results:**

Of 2085 participants without diabetes, 1208 (57.9%) were *H. pylori* seropositive in 2002. After multivariate adjustment of possible diabetes risk factors, *H. pylori* seropositivity was associated with lower risk of diabetes (RR 0.78 [95% CI 0.63, 0.97], *p* = 0.022). Of the 1275 participants with *H. pylori* antibody measurements in both 2002 and 2007, 677 (53.1%) were persistently seropositive. A lower risk of diabetes was also observed in participants with persistent *H. pylori* seropositivity (RR 0.61 [95% CI 0.41, 0.93], *p* = 0.020), compared with those persistently seronegative.

**Conclusions/interpretation:**

*H. pylori* seropositivity was associated with lower risk of diabetes in this prospective cohort study. Extrapolation of these results and the mechanism underlying the observed association require further investigation.

**Electronic supplementary material:**

The online version of this article (10.1007/s00125-017-4465-2) contains peer-reviewed but unedited supplementary material, which is available to authorised users.

## Introduction

It is estimated that more than 50% of the world’s population are infected with *Helicobacter pylori*, a gram-negative bacterium primarily colonising the human stomach [1]. The harmful effects of *H. pylori* infection on several gastrointestinal diseases have been well established [2]. An increasing number of studies have also revealed that *H. pylori* infection may have a wider ranging impact on health through its extra-gastrointestinal effects [3]. Specifically, this includes an association between *H. pylori* infection and diabetes [4–6], a harmful metabolic disease with rapidly increasing prevalence worldwide, particularly over the past 30 years [7].

A large number of published studies, reviews and book chapters have reported associations between *H. pylori* infection and serum or gastric concentrations of various hormones [2, 8–10], such as insulin, glucagon-like peptide 1, leptin, ghrelin, gastrin and somatostatin, some of which are involved in glucose metabolism directly or indirectly [11–17]. These studies provided the preliminary pathophysiological evidence for the potential connection between *H. pylori* infection and diabetes risk. Several studies have reported direct evidence for the association between *H. pylori* infection and diabetes risk [4–6]. However, most of these studies were based on relatively small sample sizes using either a cross-sectional or case–control design, with inconsistent results.

Whether *H. pylori* infection is indeed associated with diabetes risk is critically important and relevant to clinical decisions regarding future treatment of *H. pylori* as well as diabetes prevention strategies. Therefore, the purpose of this study was to examine the association between *H. pylori* infection and the long-term risk of developing diabetes based on a prospective cohort study with a relatively large sample size and multiple *H. pylori* test results.

## Methods

### Study population

Study participants were recruited from the Chinese Multi-provincial Cohort Study; a community-based cohort study from 1992 [18]. Initially, 2349 of 2505 participants without diabetes, from two communities in Beijing, China, with blood samples obtained in 2002 for *H. pylori* measurement were enrolled in this study. We excluded 45 (1.9%) deaths not related to diabetes and 219 (9.3%) participants who were lost to follow-up. Ultimately, data from 2085 (88.8%) individuals that had participated in either the 2007 (*n* = 1945) or 2012 (*n* = 1654) survey for diabetes were eligible for analysis to explore the association between *H. pylori* and the 10 year risk of developing diabetes (2002–2012; ESM Fig. 1a). Among these 2085 participants, 1728 had blood samples collected in 2007 for a second measurement of *H. pylori* antibodies. To evaluate the association between persistent *H. pylori* seropositivity (defined as participants testing seropositive to *H. pylori* in both 2002 and 2007) and risk of diabetes in 2012, 1275 participants without diabetes in both 2002 and 2007 with two *H. pylori* testing results were selected (ESM Fig. 1b).

The baseline (2002) characteristics of the recruited participants and participants lost to follow-up were compared (ESM Table 1). There was no statistically significant difference in the *H. pylori* seropositivity rate between the two groups.

The study was approved by the Ethics Committee of Beijing Anzhen Hospital. Participants provided written informed consent during the 2002, 2007 and 2012 surveys.

### Establishment of *H. pylori* serostatus

*H. pylori* antibody concentrations were evaluated using previously frozen (−80°C) serum samples obtained in 2002 and 2007 without freeze-thaw cycles. We measured serum *H. pylori* antibodies in samples collected in 2002 from all 2085 participants. Among the 2085 participants, 1275 participants had samples available for further measurement of *H. pylori* antibodies in 2007. All *H. pylori* antibodies were measured by latex-enhanced turbidimetric immunoassay (Denka Seiken, Tokyo, Japan). *H. pylori* seropositivity was defined as *H. pylori* antibodies ≥ 10 U/ml with a sensitivity of 94.0% and specificity of 91.7% when using the endoscopic gastric mucosal atrophy and rapid urease test as the gold standard [19]. Persistent seropositivity was defined as *H. pylori* antibodies ≥ 10 U/ml in both the 2002 and 2007 samples for the same person.

Pre-study validation of the *H. pylori* antibody assay was performed by measuring *H. pylori* antibody in four quality control serum samples of known low-concentrations and four known high-concentrations each day for 5 consecutive days (ESM Fig. 2). Variation in detection of *H. pylori* antibodies was small in the pre-study. Alongside measurements of *H. pylori* antibody in the study population, six quality control serum samples were added at different positions in each batch, including three known low- and three known high-concentration *H. pylori* antibody samples. The mean coefficient of variation was 9.98% for the low-concentration *H. pylori* antibody controls and 3.26% for the high-concentration *H. pylori* antibody controls. All measurements were completed using the automatic biochemical analyser (Hitachi 7180, Hitachi, Tokyo, Japan).

### Establishment of diabetes status

Information regarding diabetes status was obtained from interview during on-site surveys, fasting serum glucose measurements on the 2007 or 2012 survey day and death reports during the follow-up period. The surveys in 2007 and 2012 were conducted face-to-face by specially trained investigators. Whether individuals had received a formal diagnosis of diabetes by a physician and whether the individuals had used glucose-lowering medications during the past 2 weeks was recorded in a standardised questionnaire. Fasting glucose concentrations were measured on fresh overnight fasting blood samples on the day of serum collection during both the 2002 and 2007 surveys by enzymatic methods (2002: Daiichi Pure Chemicals, Tokyo, Japan; 2007: HUMAN, Wiesbaden, Germany). Deaths were identified at follow-up visits by staff from collaborating centres and by regular searching of the death registration database of Beijing, which includes all deaths in the Beijing population. Diabetes was defined as any of the following criteria: (1) self-report of a physician’s diagnosis of diabetes during the face-to-face survey, (2) use of glucose-lowering medications during the past 2 weeks, (3) fasting blood glucose (FBG) ≥ 7.0 mmol/l or (4) diabetes listed as the underlying cause of death on a death report.

### Assessment of relevant covariates

Other information, including demographic variables, level of education, smoking status and personal medical history, was collected in 2002 and 2007 [20]. Level of education was divided into two categories according to whether the participants received a college education (education time ≤ 12 or > 12 years). Smoking was defined as smoking one or more cigarettes per day for at least 3 months. Lipid-lowering therapies were recorded based on whether the participants were taking lipid-lowering medication.

Physical examinations, including height, weight and BP were performed by trained physicians during the on-site surveys in 2002 and 2007. BMI was calculated as weight in kilograms divided by height squared in meters. BP was measured with a regular mercury sphygmomanometer on the right arm while seated after resting for at least 5 min. The mean value of two (2002) or three (2007) consecutive readings was used. Hypertension was defined as systolic BP ≥ 140 mmHg, diastolic BP ≥ 90 mmHg and/or current antihypertensive treatment.

Laboratory measurements, including total cholesterol, triacylglycerol, LDL-cholesterol (LDL-C) and HDL-cholesterol (HDL-C) levels, were measured using fresh samples on the same day as serum collection in 2002 or 2007. Total cholesterol and triacylglycerol levels were determined using enzymatic methods (2002: Daiichi Pure Chemicals; 2007: HUMAN). LDL-C and HDL-C levels were measured using a homogeneous assay (2002: Daiichi Pure Chemicals; 2007: HUMAN). High-sensitivity C-reactive protein (Hs-CRP) was measured in 2015 using serum obtained in 2002 and 2007 by a latex-enhanced turbidimetric immunoassay assay (Denka Seiken) on an automatic biochemical analyser (Hitachi 7180).

### Sample power estimation

One cohort study has investigated the association between *H. pylori* seropositivity and risk of diabetes [6]. This study reported that those who were seropositive for *H. pylori* at recruitment were 2.69 times to develop diabetes than seronegative individuals. In our study, the cumulative incidence of diabetes in 10 years was 13.6% in seronegative participants. Based on this information with an alpha (probability of type I error) of 0.05, the actual sample size of 2085 in this study provided sufficient statistical power (power = 0.86).

### Statistical analysis

Baseline characteristics of the participants are described as mean (SD) for continuous variables with a normal distribution, median (interquartile range, IQR) for continuous variables with a skewed distribution and percentages for categorical variables. Baseline characteristics of the participants with and without *H. pylori* seropositivity were compared using the *t* test, Wilcoxon rank test or *χ* ^2^ test.

To examine the association between *H. pylori* seropositivity and diabetes risk (2002–2012) or persistent *H. pylori* seropositivity and diabetes risk (2007–2012), modified Poisson regression was performed to estimate RR and robust standard errors to estimate the 95% CIs [21]. Univariate analysis was performed first. Then, we performed a sex- and age-adjusted model (Model 1). We added further possible risk factors of diabetes to evaluate the association (Model 2), including BMI (continuous variable), HDL-C (continuous variable), triacylglycerol (< 1.7 or ≥ 1.7 mmol/l), FBG (continuous variable), systolic blood pressure (per 5 mmHg, continuous variable) and lipid-lowering medication (no or yes). Hs-CRP (< 3 or ≥ 3 mg/l), a sensitive biomarker of inflammation, reported to be associated with high risk of diabetes [22], was added in Model 3. Moreover, level of education was also included in the model (Model 4), since socioeconomic status was an important factor for *H. pylori* infection, and level of education was associated with socioeconomic status.

Since participants did not undergo OGTT at baseline, the study may have included participants with diabetes only detectable by OGTT, particularly among participants with impaired fasting glucose (IFG) [23]. Thus, we further excluded participants with IFG (FBG > 6.1 mmol/l) from the sensitivity analysis.

Subgroup analyses were performed using baseline (2002) characteristics, including age (< 60 or ≥ 60 years), sex (men or women), BMI (< 25 or ≥ 25 kg/m^2^), HDL-C (< 1.0 or ≥ 1.0 mmol/l), triacylglycerol (< 1.7 or 1.7 mmol/l), Hs-CRP (< 3 or ≥ 3 mg/l), hypertension (no or yes), FBG (< 5.6 or ≥ 5.6 mmol/l) and lipid-lowering therapy (no or yes) in a multivariable adjusted Poisson regression model. Alongside the grouping factors, other factors were used as adjustment variables. In addition, we performed further subgroup analyses using different baseline information (2002 and 2007) for the 5 year association between *H. pylori* and risk of diabetes. RRs between subgroups were compared using *Z*-test [24].

Statistical analyses were conducted by SAS 9.2 (SAS Institute, Cary, NC, USA). Two-tailed *p* values < 0.05 were considered statistically significant.

## Results

### Baseline characteristics of the study participants

The mean age of the participants (*n* = 2085) was 57.0 (±7.8) years in 2002 and 1214 (58.2%) participants were women. The *H. pylori* seropositivity rate was 57.9% in 2002. Baseline (2002) characteristics of the study participants were compared according to *H. pylori* serostatus (Table 1). *H. pylori* seropositive participants were younger than seronegative participants and had a significantly higher BMI and lower serum HDL-C and a lower proportion had hypertension. The proportion of participants receiving college education (≥ 12 years) was higher among the *H. pylori* seronegative group. There were no significant differences in other characteristics between the *H. pylori* seropositive and seronegative groups.

**Table 1:** Baseline (2002) characteristics of the study participants

	*H. pylori* seronegativity (*n* = 877)	*H. pylori* seropositivity (*n* = 1208)	*p* value
Age, mean (SD), years	58.0 (7.8)	56.3 (7.8)	< 0.001
Women, *n* (%)	509 (58.0)	705 (58.4)	0.883
BMI, mean (SD), kg/m^2^	25.0 (3.2)	25.4 (3.3)	0.005
Blood lipid levels, mean (SD), mmol/l			
Total cholesterol	5.4 (1.0)	5.3 (1.0)	0.060
LDL-C	3.3 (0.8)	3.2 (0.8)	0.373
HDL-C^a^	1.41 (0.33)	1.36 (0.30)	< 0.001
Triacylglycerol, median (IQR)	1.3 (0.9–1.9)	1.3 (0.9–1.9)	0.775
FBG, mean (SD), mmol/l	4.7 (0.5)	4.7 (0.5)	0.578
BP, mean (SD), mmHg			
Systolic	130.5 (19.2)	129.4 (18.4)	0.204
Diastolic	81.7 (10.1)	81.8 (10.2)	0.765
Hs-CRP, median (IQR), mg/l	1.1 (0.6–2.2)	1.1 (0.6–2.2)	0.225
Current smoking, *n* (%)	113 (12.9)	143 (11.8)	0.472
Hypertension, *n* (%)	416 (47.4)	495 (41.0)	0.003
Lipid-lowering medication, *n* (%)	67 (7.6)	82 (6.8)	0.456
Education ≤ 12 years, *n* (%)	342 (39.0)	526 (42.5)	0.038

^a^There is a statistical difference in HDL-C between *H. pylori* seronegative and *H. pylori* seropositive groups. We have retained two decimal places for this data to make this difference clear

### Association between *H. pylori* seropositivity and risk of developing diabetes

During the follow-up period (2002–2012), 259 (12.4%) new cases of diabetes were recorded. Among these participants, 140 (11.6%) were identified in the *H. pylori* seropositive group and 119 (13.6%) were in the seronegative group. Table 2 displays the RRs and 95% CIs for the association between *H. pylori* seropositivity and diabetes risk. Using univariate analysis, *H. pylori* was associated with a reduced risk of diabetes (RR 0.85 [95% CI 0.68, 1.07]) but without statistical significance (*p* = 0.176). The RR value was almost unchanged after sex- and age-adjustment (RR 0.86 [95% CI 0.68, 1.08], *p* = 0.200). After multivariate adjustment for possible diabetes risk factors, *H. pylori* seropositivity was associated with a significantly lower risk of diabetes (RR 0.78 [95% CI 0.63, 0.97], *p* = 0.025). This RR value was unchanged after further adjustment for Hs-CRP (RR 0.78 [95% CI 0.63, 0.97], *p* = 0.025) and level of education (RR 0.78 [95% CI 0.63, 0.97]; *p* = 0.022).

**Table 2:** Association between *H. pylori* infection and risk of developing diabetes (2002–2012)

	All participants (*n* = 2085)		Participants without IFG (*n* = 2043)	
	RR (95% CI)	*p* value	RR (95% CI)	*p* value
Events No.	259		235	
Unadjusted	0.85 (0.68, 1.07)	0.176	0.86 (0.67, 1.09)	0.215
Model 1	0.86 (0.68, 1.08)	0.200	0.86 (0.67, 1.10)	0.223
Model 2	0.78 (0.63, 0.97)	0.025	0.80 (0.64, 1.01)	0.059
Model 3	0.78 (0.63, 0.97)	0.025	0.79 (0.63, 0.99)	0.043
Model 4	0.78 (0.63, 0.97)	0.022	0.80 (0.64, 1.01)	0.057

Model 1: Sex- and age-adjusted

Model 2: Model 1 + BMI (continuous variable), systolic BP (per 5 mmHg, continuous variable), HDL-C (continuous variable), triacylglycerol (< 1.7 or ≥ 1.7 mmol/l), FBG (continuous variable), lipid-lowering therapy (no or yes)

Model 3: Model 2 + Hs-CRP (< 3 or ≥ 3 mg/l)

Model 4: Model 3 + level of education (≤ 12 or > 12 years)

No., number

After further excluding participants with IFG, the association between *H. pylori* and diabetes risk was similar (Table 2; details in ESM Table 2).

### Association between persistent *H. pylori* seropositivity and risk of developing diabetes

A total of 1275 participants had *H. pylori* antibody measurements in both 2002 and 2007, of which 677 (53.1%) were persistently seropositive and 514 (40.3%) were persistently seronegative. Only 19 (1.5%) participants transformed from seronegativity to seropositivity and 65 (5.1%) from seropositivity to seronegativity. A total of 84 individuals developed diabetes among these participants between 2007 and 2012, including 44 (8.6%) among those who were persistently seronegative, 35 (5.2%) among those who were persistently seropositive and five (7.7%) among those who transformed from seropositive to seronegative. No new cases of diabetes were identified among those transformed from seronegative to seropositive. As the serostatus only changed in a very small number of participants, we compared the risk of developing diabetes in participants who were persistently seropositive with those who were persistently seronegative, with 2007 as the baseline. Therefore, 1191 participants were included in the analysis of the association between persistent *H. pylori* seropositivity and diabetes risk. The baseline (2007) characteristics of these participants were compared across *H. pylori* serostatus (ESM Table 3). As displayed in Table 3 (details in ESM Table 4), persistent *H. pylori* seropositivity was also associated with a lower risk of developing diabetes in both univariate and multivariate analyses. After excluding participants with IFG, *H. pylori* seropositivity was still associated with lower risk of diabetes (Table 3).

**Table 3:** Association between persistent *H. pylori* infection and risk of developing diabetes (2007–2012)

	All participants (*n* = 1191)		Participants without IFG (*n* = 1036)	
	RR (95% CI)	*p* value	RR (95% CI)	*p* value
Events No.	79		37	
Unadjusted	0.60 (0.39, 0.93)	0.021	0.52 (0.27, 0.98)	0.044
Model 1	0.58 (0.37, 0.89)	0.013	0.50 (0.26, 0.98)	0.044
Model 2	0.62 (0.41, 0.92)	0.019	0.50 (0.26, 0.99)	0.047
Model 3	0.61 (0.41, 0.91)	0.017	0.51 (0.25, 1.01)	0.054
Model 4	0.61 (0.41, 0.93)	0.020	0.50 (0.26, 0.99)	0.048

Persistent seropositivity: individuals were seropositive in both 2002 and 2007

Model 1: Sex- and age-adjusted

Model 2: Model 1 + BMI (continuous variable), systolic BP (per 5 mmHg, continuous variable), HDL-C (continuous variable), triacylglycerol (< 1.7 or ≥ 1.7 mmol/l), FBG (continuous variable), lipid-lowering therapy (no or yes)

Model 3: Model 2 + Hs-CRP (< 3 or ≥ 3 mg/l)

Model 4: Model 3 + level of education (≤ 12 or > 12 years)

No., number

### Subgroup analysis for the association between *H. pylori* seropositivity and risk of developing diabetes

**Fig. 1:**
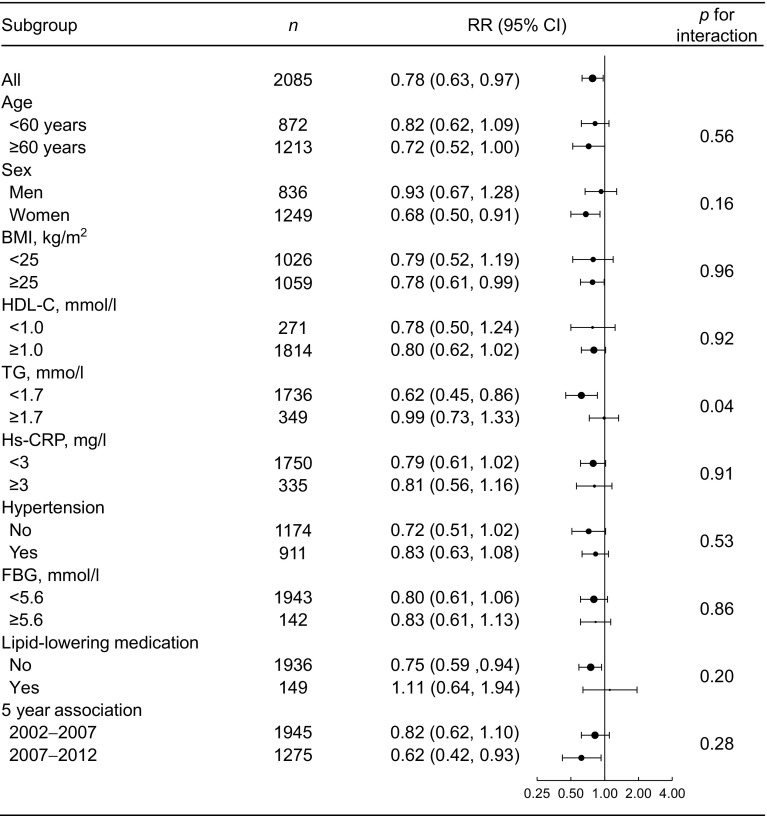
Subgroup analyses for the association between *H. pylori* seropositivity and risk of developing diabetes. All RRs were adjusted for age (continuous variable), sex (men or women), BMI (continuous variable), systolic BP (per 5 mmHg, continuous variable), HDL-C (continuous variable), triacylglycerol (< 1.7 or ≥ 1.7 mmol/l), FBG (continuous variable), lipid-lowering therapy (no or yes) and Hs-CRP (< 3 or ≥ 3 mg/l), except for the grouping variable

The associations between *H. pylori* seropositivity and risk of developing diabetes were evaluated based on different subgroups, including age (< 60 or ≥ 60 years), sex (men or women), BMI (< 25 or ≥ 25 kg/m^2^), HDL-C (< 1.0 or ≥ 1.0 mmol/l), triacylglycerol (< 1.7 or ≥ 1.7 mmol/l), Hs-CRP (< 3 or ≥ 3 mg/l), hypertension (no or yes), FBG (< 5.6 or ≥ 5.6 mmol/l) and lipid-lowering therapy (no or yes) (Fig. 1). *H. pylori* seropositivity was associated with lower risk of developing diabetes in different subgroups except in participants receiving lipid-lowering medication (RR 1.11 [95% CI 0.64, 1.94]). In addition, *H. pylori* seropositivity was associated with a lower 5 year risk of developing diabetes based on different baseline information (2002–2007; 2007–2012).

## Discussion

In this prospective cohort study, we carefully examined the association between *H. pylori* seropositivity and risk of developing diabetes, based on reliable measurements of antibody against *H. pylori* and a clear diagnosis of diabetes. We determined that *H. pylori* seropositivity was associated with lower risk of developing diabetes. To our knowledge, this is the first cohort study to evaluate the association between persistent *H. pylori* seropositivity and risk of developing diabetes and the first study to detect an inverse association between these factors.

The literature regarding the association between *H. pylori* and diabetes risk is inconsistent, with reports of positive, null and negative associations [4–6]. A published meta-analysis systematically reviewed the association between *H. pylori* infection and diabetes based on observational studies and drew the conclusion that there was a trend toward more frequent *H. pylori* infection in individuals with diabetes [4]. However, cross-sectional or case–control studies could not determine the causality between *H. pylori* infection and diabetes. We are aware of only one prospective cohort study evaluating the association between *H. pylori* seropositivity and risk of diabetes [6]. This study derived data from the Sacramento Area Latino Study on Ageing and found that *H. pylori* seropositivity was associated with increased risk of incident diabetes (HR 2.7 [95% CI 1.1, 6.6]). However, the characteristics of our study population differed from this study in that their participants were older (60 to 101 years old) and the *H. pylori* seropositivity rate was much higher than ours (91.9% vs 57.9%).

Our finding of an inverse association between *H. pylori* seropositivity and diabetes risk was of physiological relevance. Several studies have reported that *H. pylori* seropositivity was associated with different hormones [2, 8–10], most of which were involved in glucose metabolism directly or indirectly [11–17]. To our knowledge, at least three hormones supported our results, including gastrin, leptin and ghrelin. Studies have reported that long-term gastrin treatment resulted in improved metabolic control and exerted proliferative effects on pancreatic beta cells [11]. Leptin has been reported to reduce hyperglycaemia in rodent models of type 1 diabetes and has recently been shown to normalise fasting plasma glucose concentrations in a rodent model of polygenic obesity and type 2 diabetes [14]. In contrast, ghrelin has been shown to upregulate systemic glucose levels in both humans and rodents [16]. Thus, studies reporting elevated gastrin and leptin and decreased ghrelin levels in individuals infected with *H. pylori* support our findings. However, the regulation of these hormones is intricate. Therefore, the association between *H. pylori* infection, hormones and diabetes requires further studies.

*H. pylori* has co-existed with humans for at least 58,000 years [25], and only 10% of asymptomatic infected individuals develop gastrointestinal disease during their lifetime [1]. Regarding the view of co-adaptation of humans and microorganisms, we might reconsider our definitions of *H. pylori* and perhaps recognise it as a normal member of the human gastric microbiome [26]. The Kyoto global consensus on *H. pylori* gastritis, published in 2015, strongly recommended that all individuals infected with *H. pylori* should be offered eradication therapy, unless there were competing considerations [27]. If *H. pylori* infection could indeed affect diabetes risk, we should reconsider this strategy. More studies are needed to explore the association between these factors.

Several limitations associated with the current study deserve mention. First, 45 participants died and 219 were lost at follow-up between 2002 and 2012, which raised a consideration for the competing risk. To test each extreme situation, we analysed the association between *H. pylori* infection and risk of developing diabetes assuming that all of the participants who died or were lost at follow-up did not develop diabetes and then again, but assuming that all did develop diabetes (ESM Table 5). In both of these analyses, *H. pylori* seropositivity was still associated with lower risk of diabetes. Therefore, we believe that loss and death in our study have little effect on our findings. Additionally, time-to-event analysis could not be performed in our study as we only collected information regarding the development of diabetes at 5 yearly intervals. Furthermore, we did not collect the information about *H. pylori* eradication treatments in our study. However, as we knew, the awareness rate of *H. pylori* was very low even among individuals with gastrointestinal symptoms in China [28], so we could speculate that the treatment rate could also be very low. In addition, we detected *H. pylori* in both 2002 and 2007 and found that the serostatus changed in only a few participants. To some extent, multi-point detection might better explain the long-term relationship between *H. pylori* and humans. In addition, we did not specifically measure the concentrations of antibodies against cytotoxin-associated gene A (CagA), a major factor influencing the virulence of *H. pylori*, in our population. A study has reported that FBG levels were significantly higher in uninfected mice compared with *H. pylori*-infected mice, regardless of the strain of *H. pylori* utilised [29]. However, only mice infected with a Cag pathogenicity island (PAI)-negative *H. pylori* strain, but not with an isogenic Cag PAI-positive strain, showed improvements in glucose tolerance. Therefore, further studies are still needed to investigate the association between antibodies against CagA and glycometabolism.

In summary, these findings provide new direct evidence for the association between *H. pylori* infection and diabetes risk. Analysis of the association between *H. pylori* and diabetes is of great public health and clinical significance given the high prevalence of *H. pylori* infection and significant burden of diabetes. Thus, clarification of the association between these factors might influence clinical decisions regarding future treatment of *H. pylori* infection as well as diabetes prevention strategies. However, extrapolation of this study requires verification by other prospective studies and clinical trials, and the underlying mechanism warrants further investigation.

## Electronic supplementary material

ESM (PDF 317 kb)

## Abbreviations

CagA: Cytotoxin-associated gene A
FBG: Fasting blood glucose
HDL-C: HDL-cholesterol
Hs-CRP: High-sensitivity C-reactive protein
IFG: Impaired fasting glucose
IQR: Interquartile range
LDL-C: LDL-cholesterol
PAI: Pathogenicity island

## References

[1] McColl KE (2010). *Helicobacter pylori* infection. N Engl J Med.

[2] Hidekazu S, Robin W, Barry M (2016). Helicobacter pylori.

[3] Franceschi F, Zuccala G, Roccarina D, Gasbarrini A (2014). Clinical effects of *Helicobacter pylori* outside the stomach. Nat Rev Gastroenterol Hepatol.

[4] Zhou X, Zhang C, Wu J, Zhang G (2013). Association between *Helicobacter pylori* infection and diabetes mellitus: a meta-analysis of observational studies. Diabetes Res Clin Pract.

[5] Haj S, Raviv M, Muhsen K (2016) *Helicobacter pylori* infection and diabetes mellitus. In: Bruna MR (eds) Extradigestive manifestations of *Helicobacter pylori* infection—an overview, Dr B Roesler (Ed.), InTech, 10.5772/63826

[6] Jeon CY, Haan MN, Cheng C (2012). *Helicobacter pylori* infection is associated with an increased rate of diabetes. Diabetes Care.

[7] Zhou B, Lu Y, Hajifathalian K (2016). Worldwide trends in diabetes since 1980: a pooled analysis of 751 population-based studies with 4.4 million participants. Lancet.

[8] Polyzos SA, Kountouras J, Zavos C, Deretzi G (2011). The association between *Helicobacter pylori* infection and insulin resistance: a systematic review. Helicobacter.

[9] Yap TW, Leow AH, Azmi AN (2015). Changes in metabolic hormones in Malaysian young adults following *Helicobacter pylori* eradication. PLoS One.

[10] Kaneko H, Konagaya T, Kusugami K (2002). *Helicobacter pylori* and gut hormones. J Gastroenterol.

[11] Tellez N, Montanya E (2014). Gastrin induces ductal cell dedifferentiation and beta-cell neogenesis after 90% pancreatectomy. J Endocrinol.

[12] Sliwinska-Mosson M, Vesely M, Milnerowicz H (2014). The clinical significance of somatostatin in pancreatic diseases. Ann Endocrinol-Paris.

[13] Dezaki K (2013). Ghrelin function in insulin release and glucose metabolism. Endocr Dev.

[14] Cummings BP (2013). Leptin therapy in type 2 diabetes. Diabetes Obes Metab.

[15] Meier JJ (2012). GLP-1 receptor agonists for individualized treatment of type 2 diabetes mellitus. Nat Rev Endocrinol.

[16] Yada T, Damdindorj B, Rita RS (2014). Ghrelin signalling in beta-cells regulates insulin secretion and blood glucose. Diabetes Obes Metab.

[17] Drucker DJ (2007). The role of gut hormones in glucose homeostasis. J Clin Invest.

[18] Liu J, Hong Y, D’Agostino RB (2004). Predictive value for the Chinese population of the Framingham CHD risk assessment tool compared with the Chinese Multi-Provincial Cohort Study. JAMA.

[19] Furuta T (2016) Evaluation of the novel anti-*H. pylori* antibody detection kit for a clinical chemistry auto-analyzer. Jpn J Med Pharm Sci 73:451–457 [article in Japanese]

[20] Liu J, Wang W, Qi Y (2014). Association between the lipoprotein-associated phospholipase A2 activity and the progression of subclinical atherosclerosis. J Atheroscler Thromb.

[21] Lumley T, Kronmal R, Ma S (2006) Relative risk regression in medical research: models, contrasts, estimators, and algorithms. UW Biostatistics Working Paper Series. Working paper 293. Available from http://biostats.bepress.com/uwbiostat/paper293

[22] Wang X, Bao W, Liu J (2013). Inflammatory markers and risk of type 2 diabetes: a systematic review and meta-analysis. Diabetes Care.

[23] Liu J, Zhao D, Liu J, Qi Y, Sun J, Wang W (2013). Prevalence of diabetes mellitus in outpatients with essential hypertension in China: a cross-sectional study. BMJ Open.

[24] Altman DG, Bland JM (2003). Interaction revisited: the difference between two estimates. BMJ.

[25] Linz B, Balloux F, Moodley Y (2007). An African origin for the intimate association between humans and *Helicobacter pylori*. Nature.

[26] Atherton JC, Blaser MJ (2009). Coadaptation of *Helicobacter pylori* and humans: ancient history, modern implications. J Clin Invest.

[27] Sugano K, Tack J, Kuipers EJ (2015). Kyoto global consensus report on *Helicobacter pylori* gastritis. Gut.

[28] Dong J (2015). Gastroscopy for Hpylori infection awareness survey analysis. China Health Ind 2015.

[29] Bassaganya-Riera J, Dominguez-Bello MG, Kronsteiner B (2012). *Helicobacter pylori* colonization ameliorates glucose homeostasis in mice through a PPAR gamma-dependent mechanism. PLoS One.

