# Supplementary material for: The association between Helicobacter pylori seropositivity and risk of new-onset diabetes: a prospective cohort study
Source: Diabetologia. 2017 Oct 30;61(2):300–7. doi: 10.1007/s00125-017-4465-2 (PMC6448956; doi:10.1007/s00125-017-4465-2)
Supplement: Supplementary file 1 — (PDF 317 kb) [file 125_2017_4465_MOESM1_ESM.pdf]

Electronic Supplementary Material

Contents

Page 2. ESM Table 1 Baseline characteristics of the participants who were included and who were lost in the follow-up (2002-2012)

Page 3. ESM Table 2 Multivariate analysis of the association between *H. pylori* seropositivity and risk of developing diabetes (2002–2012)

Page 4. ESM Table 3 Baseline (2007) characteristics of the study participants

Page 5. ESM Table 4 Multivariate analysis of the association between persistent *H. pylori* seropositivity and risk of developing diabetes (2007–2012)

Page 6. ESM Table 5 Competing risk analysis for the participants lost and died (2002-2012)

Page 7-8. ESM Fig. 1 Flow chart of study population

Page 9. ESM Fig. 2 Pre-Study validation of the *H. pylori* antibodies measurement with known concentrations of *H. pylori* antibodies samples.

ESM Table 1 Baseline characteristics of the participants who were included and who were lost in the follow-up (2002-2012)

	Participants eligible (*n*=2085)	Participants lost (*n*=219)	*p* value
H. pylori seropositivity	1208 (58.0)	116 (53.0)	0.157
Age, mean (SD), years	57.0 (7.8)	58.4 (8.3)	0.014
Women (%)	1214 (58.2)	99 (45.2)	<0.001
BMI, mean (SD), kg/m^2	25.2 (3.3)	25.4 (3.4)	0.502
Blood lipid levels, mean (SD), mmol/l			
TC	5.4 (1.0)	5.3 (1.0)	0.166
LDL-C	3.2 (0.8)	3.3 (0.8)	0.815
HDL-C	1.4 (0.3)	1.3 (0.3)	0.022
TG	1.3 (0.9-1.9)	1.4 (1.0-2.0)	0.220
FBG, mean (SD), mmol/l	4.7 (0.5)	4.7 (0.6)	0.934
Blood pressure, mean (SD), mmHg			
Systolic	129.9 (18.7)	133.0 (20.3)	0.023
Diastolic	81.8 (10.2)	82.9 (10.6)	0.119
Hs-CRP, median (IQR),mg/l	1.1 (0.6-2.2)	1.3 (0.7-2.3)	0.093
Current smoking (%)	256 (12.3)	38 (17.4)	0.032
Hypertension (%)	911 (43.7)	103 (49.3)	0.111
Lipid-lowering medication (%)	149 (7.1)	11(5.0)	0.240
Education ≤ 12 years (%)	869 (41.7)	100 (45.7)	0.252

ESM Table 2 Multivariate analysis of the association between *H. pylori* seropositivity and risk of developing diabetes (2002–2012)

	All Participants (n=2085)		Participants without IFG (n=2043)	
Variables	RR (95% CI)	p value	RR (95% CI)	p value
H. pylori seropositivity	0.78 (0.63, 0.97)	0.022	0.80 (0.64, 1.01)	0.057
Age	0.99 (0.97, 1.00)	0.055	0.98 (0.97, 1.00)	0.089
Female	1.02 (0.80, 1.29)	0.709	1.03 (0.80, 1.32)	0.818
BMI	1.06 (1.03, 1.10)	<0.001	1.07 (1.04, 1.11)	<0.001
HDL-C	0.56 (0.35, 0.89)	0.015	0.53 (0.33, 0.86)	0.009
TG	1.21 (0.95, 1.55)	0.149	1.14 (0.88, 1.48)	0.327
Hs-CRP	1.46 (1.14, 1.88)	0.004	1.54 (1.19, 2.00)	0.001
SBP (5 mmHg)	1.03 (1.00, 1.06)	0.061	1.03 (0.99, 1.06)	0.108
FBG	3.38 (2.92, 3.92)	<0.001	4.52 (3.62, 5.65)	<0.001
Lipid-lowering medication	1.67 (1.23, 2.29)	0.001	1.60 (1.15, 2.23)	0.006
Education > 12 years	1.19 (0.93, 1.53)	0.158	1.17 (0.90, 1.51)	0.236

Adjusted variables: age (continuous variable), female (male or female), body mass index (continuous variable), systolic blood pressure (per 5 mmHg, continuous variable), high-density lipoprotein cholesterol (continuous variable), triacylglycerol (<1.7 or ≥1.7 mmol/l), fasting blood glucose (continuous variable), lipid-lowering therapy (no or yes), high-sensitivity C-reactive protein (<3 or ≥3 mg/l) and education levels (≤12 or >12 years)

ESM Table 3 Baseline (2007) characteristics of the study participants

	persistent *H. pylori* seronegativity (*n*=514)	persistent *H. pylori* seropositivity (*n*=677)	*p* value
Age, mean (SD), years	62.6 (7.8)	60.6 (7.7)	<0.001
Women (%)	308 (59.9)	417 (61.6)	0.558
BMI, mean (SD), kg/m^2	24.4 (3.0)	25.1 (3.4)	<0.001
Blood lipid levels, mean (SD), mmol/l			
TC	5.3 (1.0)	5.4 (1.0)	0.102
LDL-C	3.3 (0.9)	3.5 (0.8)	0.008
HDL-C	1.4 (0.3)	1.4 (0.3)	0.562
TG	1.3 (0.9-1.9)	1.3 (0.9-1.9)	0.807
FBG, mean (SD), mmol/l	5.5 (0.5)	5.5 (0.5)	0.138
Blood pressure, mean (SD), mmHg			
Systolic	135.3 (17.8)	134.4(16.7)	0.357
Diastolic	82.0 (9.7)	81.8 (9.3)	0.701
Hs-CRP, median (IQR), mg/l	1.1 (0.6-2.2)	1.1 (0.6-2.2)	0.882
Current smoking (%)	54 (10.5)	69 (10.1)	0.860
Hypertension (%)	285 (55.5)	340 (50.2)	0.074
Lipid-lowering medication (%)	24 (4.7)	26 (3.8)	0.480
Education≤12 years (%)	203 (39.5)	320 (47.3)	0.007

ESM Table 4 Multivariate analysis of the association between persistent *H. pylori* seropositivity and risk of developing diabetes (2007–2012)

	All Participants (n=1191)		Participants without IFG (n=1036)	
Variables	RR (95% CI)	p value	RR (95% CI)	p value
H. pylori seropositivity	0.61 (0.41, 0.93)	0.020	0.50 (0.26, 0.99)	0.048
Age	0.97 (0.94, 1.00)	0.023	0.97 (0.92, 1.01)	0.163
Female	1.11 (0.73, 1.69)	0.621	0.97 (0.46, 2.05)	0.940
BMI	1.03 (0.97, 1.09)	0.337	0.99 (0.89, 1.11)	0.903
HDL-C	0.63 (0.27, 1.48)	0.288	0.77 (0.23, 2.52)	0.662
TG	1.03 (0.67, 1.57)	0.909	0.99 (0.49, 1.98)	0.974
Hs-CRP	1.30 (0.82, 2.07)	0.267	2.18 (1.08, 4.41)	0.031
SBP	1.03 (0.98, 1.09)	0.268	1.05 (0.95, 1.15)	0.369
FBG	6.77 (4.57, 10.02)	<0.001	4.99 (1.81, 13.77)	0.002
Lipid-lowering medication	2.89 (1.32, 6.32)	0.008	3.72 (1.29, 10.72)	0.015
Education > 12 years	0.94 (0.63, 1.40)	0.772	0.89 (0.47, 1.69)	0.728

Adjusted variables: age (continuous variable), female (male or female), body mass index (continuous variable), systolic blood pressure (per 5 mmHg, continuous variable), high-density lipoprotein cholesterol (continuous variable), triacylglycerol (<1.7 or ≥1.7 mmol/l), fasting blood glucose (continuous variable), lipid-lowering therapy (no or yes), high-sensitivity C-reactive protein (<3 or ≥3 mg/l) and education levels (≤12 or >12 years)

ESM Table 5 Competing Risk Analysis for the participants lost and died (2002-2012)

	Model 1[a]		Model 2[b]	
Variables	RR (95% CI)	*p* value	RR (95% CI)	*p* value
H. pylori seropositivity	0.81 (0.66, 1.01)	0.061	0.87 (0.75, 1.00)	0.053
Age	0.98 (0.96, 1.00)	0.022	1.01 (1.00, 1.02)	0.146
Female	1.07 (0.84, 1.36)	0.576	0.82 (0.70, 0.97)	0.024
BMI	1.07 (1.03, 1.10)	<0.001	1.03 (1.00, 1.05)	0.030
HDL-C	0.61 (0.38, 0.97)	0.036	0.72 (0.53, 0.97)	0.033
TG	1.19 (0.93, 1.52)	0.166	1.14 (0.97, 1.34)	0.115
Hs-CRP	1.43 (1.11, 1.84)	0.005	1.34 (1.13, 1.59)	0.001
SBP (5 mmHg)	1.03 (1.00, 1.06)	0.099	1.02 (1.00, 1.04)	0.133
FBG	3.45 (2.96, 4.01)	<0.001	1.90 (1.70, 2.12)	<0.001
Lipid-lowering medication	1.74 (1.27, 2.39)	0.001	1.13 (0.89, 1.44)	0.313
Education > 12 years	1.26 (0.98, 1.61)	0.075	0.91 (0.77, 1.07)	0.259

[a] Assuming that all of these participants died or lost in follow-up did not develop diabetes between 2002 and 2012 (Total number of diabetes: 259)

[b] Assuming that all of these participants died or lost in follow-up developed diabetes between 2002 and 2012 (Total number of diabetes: 523)

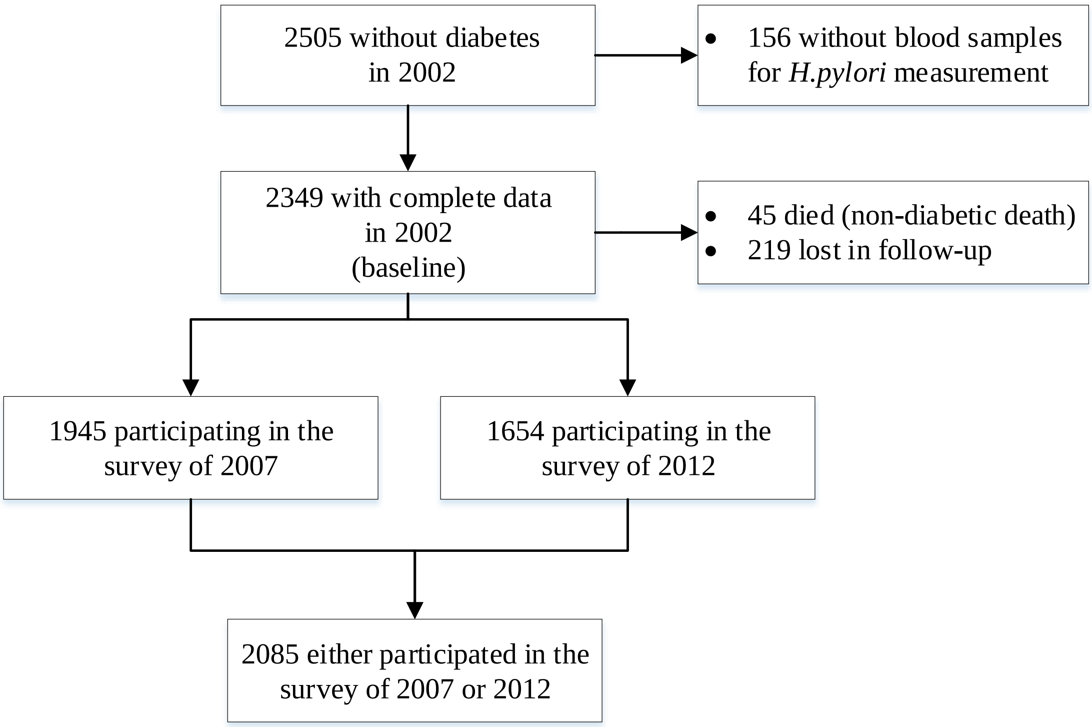

ESM Fig. 1(a) Study population

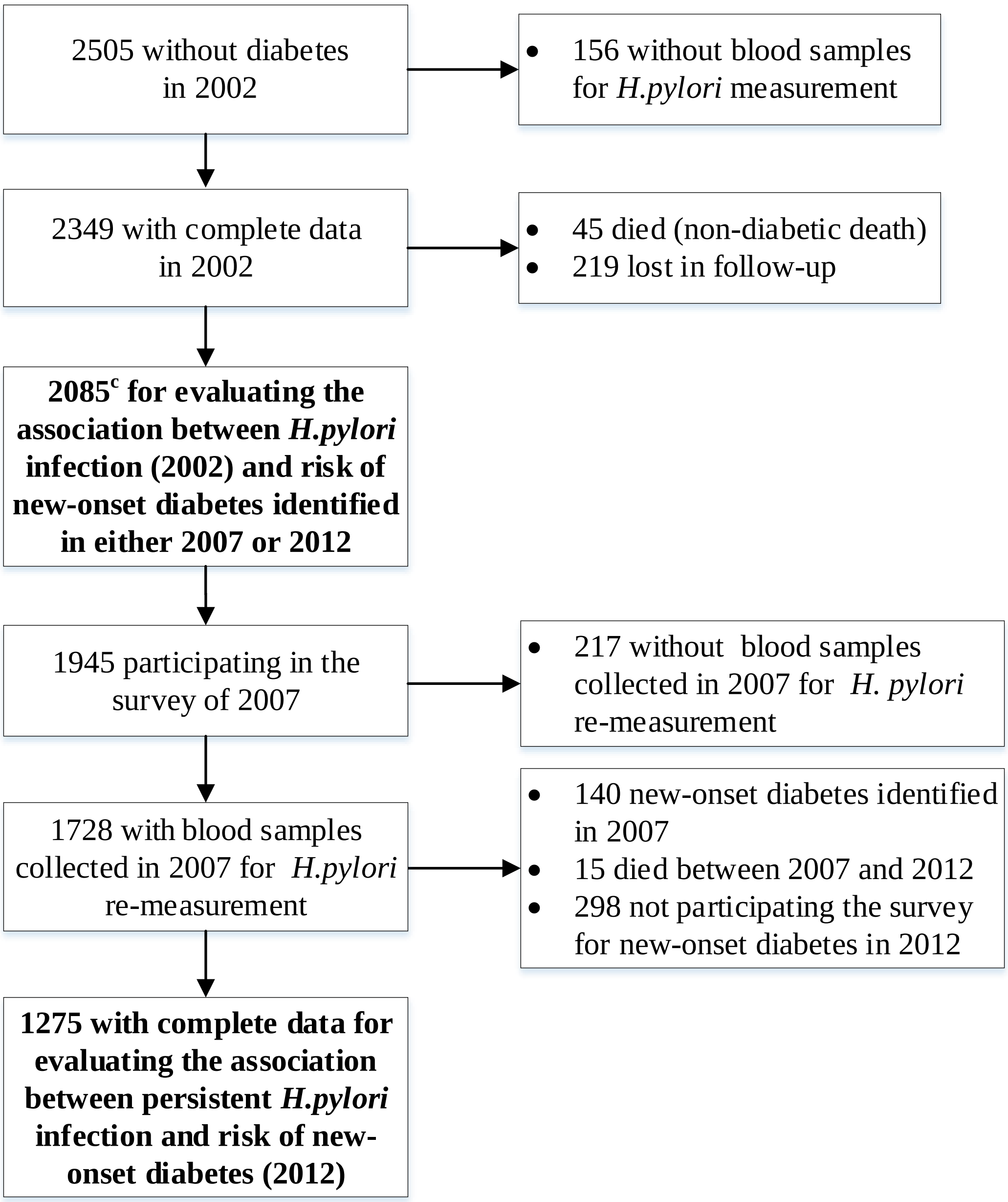

ESM Fig. 1(b) Study population

[c] One of the participants had no diabetes at baseline (2002) and did not participate in the survey of 2007, but died of diabetes in 2008. As she had complete information at baseline and had outcome, we also included her in this study.

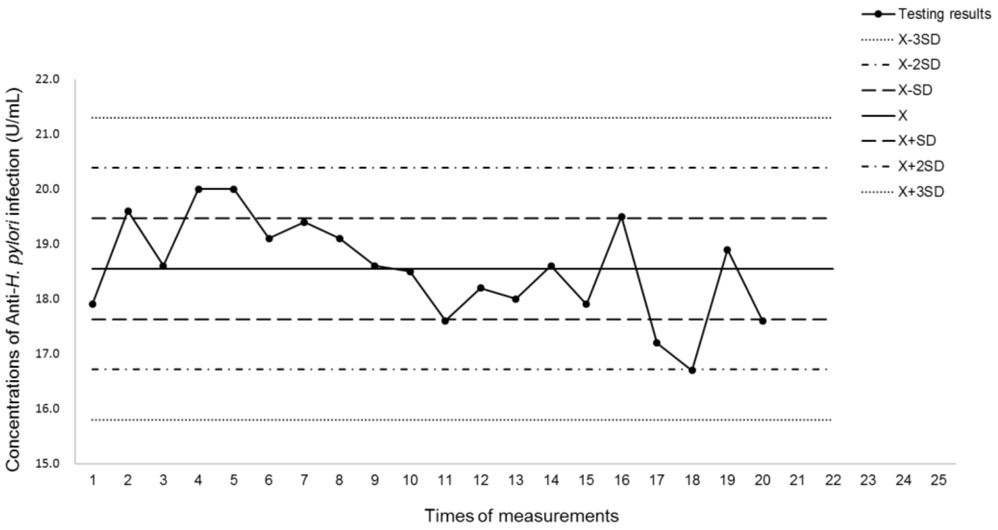

ESM Fig. 2 (a) Pre-study validation of the *H. pylori* antibodies measurement with known high-concentrations of *H. pylori* antibodies samples.

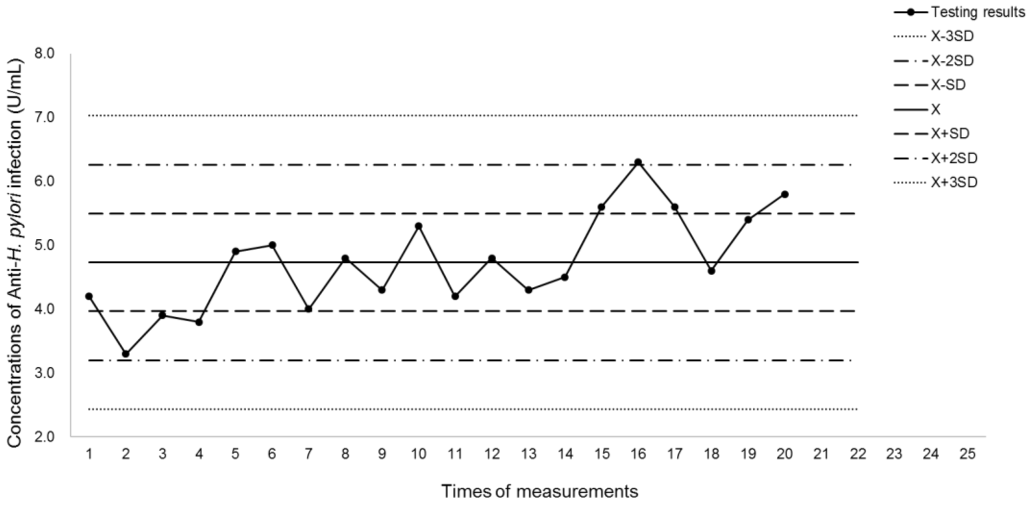

ESM Fig. 2 (b) Pre-study validation of the *H. pylori* antibodies measurement with known low-concentrations of *H. pylori* antibodies samples.
